# In Silico Modeling of Hyposalivation and Biofilm Dysbiosis in Root Caries

**DOI:** 10.1177/00220345211000655

**Published:** 2021-03-20

**Authors:** D. Head, P.D. Marsh, D.A. Devine, L.M.A. Tenuta

**Affiliations:** 1 School of Computing, University of Leeds, Leeds, UK; 2 Division of Oral Biology, School of Dentistry, Wellcome Trust Brenner Building, University of Leeds, St. James University Hospital, Leeds, UK; 3 Department of Cariology, Restorative Sciences and Endodontics, School of Dentistry, University of Michigan, Ann Arbor, MI, USA

**Keywords:** computer simulation, xerostomia, saliva, dentin, diet, cariogenic

## Abstract

Root caries progression is aggravated by hyposalivation, which can accelerate the conversion of a dental biofilm from having a symbiotic microbial relationship with the host (predominance of nonaciduric species) to a dysbiotic one (dominated by aciduric species). Using a mathematical model previously employed to investigate factors associated with biofilm dysbiosis, we systematically explored the deleterious effect of hyposalivation on the composition of the biofilm and the risk of root dentin demineralization. By varying the clearance half-times of sugar (i.e., readily fermented dietary carbohydrates), we simulated hyposalivation and investigated its effect on 1) the time that the biofilm pH spends below the minimum for dentin or enamel demineralization and 2) the conversion of the biofilm from a symbiotic to dysbiotic composition. The effect of increasing sugar clearance half-times on the time that the biofilm pH is below the threshold for demineralization was more pronounced for dentin than for enamel (e.g., increasing the clearance half-time from 2 to 6 min doubled the time that the biofilm pH was below the threshold for dentin demineralization). The effect on biofilm composition assessed at 50 d showed that the conversion from a symbiotic to a dysbiotic biofilm happened around a frequency of 6 sugar intakes per day when the clearance half-time was 2 min but only 3 sugar intakes per day when the clearance half-time was 6 min. Taken together, the results confirm the profound effect that prolonged sugar clearance has on the dynamics of dental biofilm composition and the subsequent risk of root caries. This in silico model should be applied to study how interventions that alter salivary clearance rates or modify biofilm pH can affect clinical conditions such as root caries.

## Introduction

Root caries is predicted to become more prevalent in the next decades as a result of the retention of teeth into later life (Kassebaum et al. 2014; Dye et al. 2019) and the increase in life expectancy (Kontis et al. 2017). This is driving a resurgence in research on this topic (for reviews, see Takahashi and Nyvad 2016; Tonetti et al. 2017; Meyer-Lueckel et al. 2019).

Root dentin mineral is more soluble than enamel (Hoppenbrouwers et al. 1987; Moreno and Aoba 1991), making it more likely to demineralize under milder acidic conditions. Any fall in pH caused by fermentation of sugar (the term *sugar* is used throughout this article to refer to readily fermented dietary carbohydrate, such as glucose and sucrose) is followed by a period of recovery toward neutral pH (Stephan 1944); as dentin will dissolve over a greater range of acidic pH than enamel, it will also dissolve for a longer period. Another important and perhaps more impactful factor associated with root caries is the increase in chronic use of medications that have hyposalivatory effects as people age (Hayes et al. 2016; Barbe 2018). The reduction in salivary flow drastically increases the rate of mineral dissolution because 1) sugar clearance is slowed down and therefore fermentation is prolonged; 2) there is a scarcity of buffering factors to raise the pH and provide mineral ions to replenish lost minerals; and 3) the change in local environment will drive changes in biofilm composition favoring aciduric (acid-tolerant) species (i.e., biofilm dysbiosis will occur). Moreover, older adults with hyposalivation may choose more cariogenic foods (softer, sugar rich) based on an increased oral comfort perception (Assad-Bustillos et al. 2019), or they may have other neurodegenerative disorders that decrease their oral function and increase their caries rates (Bakke et al. 2011; Cicciù et al. 2012). Surprisingly, the extent to which the progression of root caries is increased because of hyposalivation has not been fully explored in the literature, and the effect of hyposalivation on biofilm dysbiosis is only starting to be investigated in detail (Rusthen et al. 2019).

The complexity of factors associated with root caries makes it challenging to determine experimentally the significance of individual parameters to the disease process. Mathematical modeling (Dawes 1983; Ilie et al. 2012; Ilie et al. 2014; Head et al. 2014) is a valuable tool that can be used to explore the role of each factor associated with root caries development or to investigate the effect of different anticaries agents. We previously used mathematical modeling to generate complex high-fidelity representations of multispecies biofilms and studied the changes in biofilm ecology with time as a result of frequency of sugar exposure (Head et al. 2017). Here we expand this approach to facilitate a thorough investigation of the deleterious effect of variables that are relevant to hyposalivation on biofilm dysbiosis and root demineralization and to identify factors that could be the most promising targets for interventions.

## Materials and Methods

### Experimental Design

The mathematical model of Head et al. (2017) was used to assess the role of prolonged sugar clearance (employed here to simulate hyposalivation) on 1) the amount of time that the biofilm spends below the threshold for enamel or dentin demineralization and 2) the conversion of the biofilm from a symbiotic composition (comprising mainly nonaciduric species) to a dysbiotic one (comprising mainly aciduric species). Other effects of salivary dysfunction are not considered here, as investigated in previous work (e.g., buffering; Marsh et al. 2014).

### Components of the In Silico Model

**Figure 1.**
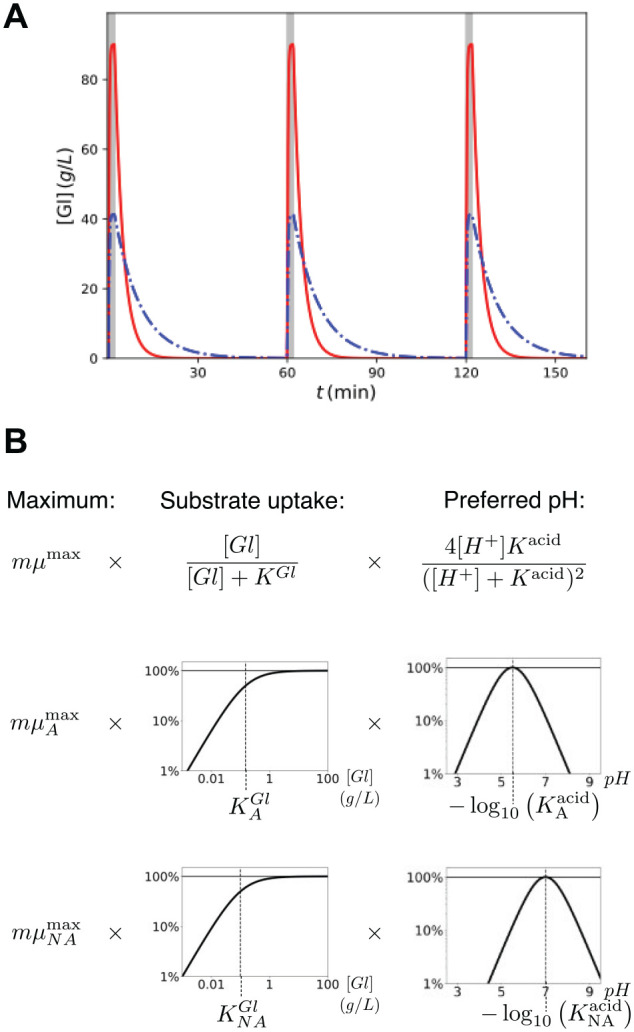
Key components of the in silico model. (**A**) Introduction of dietary glucose for *t*_½_ = 2 *min* (solid line) and 6 *min* (dot-dashed line), shown here for hourly intakes. (**B**) Graphic representation of the equations for the metabolic conversion of sugar (i.e., readily fermented dietary carbohydrates) to acid, for particle of mass *m* given local sugar concentration [*Gl*] and pH, showing the general equation (top row) and each factor for particles of type A (aciduric; middle row) and NA (nonaciduric; lower row).

The mathematical model comprised discrete spherical particles representing bacterial cell aggregates encased in an extracellular matrix and concentration fields representing dispersed phases (nutrients and metabolites). Particles were 1 of 2 types: A for aciduric (i.e., acid tolerant), capable of metabolizing sugars to acid at low pH and persisting under such conditions, and NA for nonaciduric (i.e., not acid tolerant), with metabolic rates that are sharply reduced at lowered environmental pH (de Soet et al. 2000). Dietary sugars (the limiting nutrient), denoted [*Gl*], and the undisassociated acid metabolite, [*acid*], were represented as concentration fields spanning the full system and obeying the reaction-diffusion equation with specified boundary conditions: both obeyed the zero-flux requirement at the tooth surface, and the acid concentration was zero at the saliva-air interface. The sugar concentration at the same interface was varied to represent periodic dietary intakes, rapidly increasing at the start of each episode and followed by an exponential decay with a half-decay *t*_½_ (Dawes 1983; Ilie et al. 2012; Ilie et al. 2014). The integrated sugar input was assumed to be the same for all *t*_½_, so a shorter *t*_½_ corresponded to a higher peak value, as shown in Figure 1. Keeping the integrated concentration of sugar fixed in this manner allowed the effect of the clearance time *t*_½_ to be evaluated independently of other mechanisms (Dawes 1983). Periodic boundary conditions were assumed in lateral directions. In addition, endogenous and exogenous nutrient sources present in the matrix and saliva (Bradshaw et al. 1994) were represented as a low concentration of carbohydrate, [*polyGl*], that was uniform in space and constant in time.

### Metabolic Rate and Cell Growth

The spatial profiles of [*Gl*] and [*acid*] at each time point were found by solving the coupled reaction-diffusion equations with given reaction rates of conversion of sugar to acid per particle. The rate of acid production by a particle of type A with mass *m*, in the presence of a concentration of sugar [*Gl*], obeyed the equation

(1) $$\begin{array}{c} r=m\mu_{A}^{max}\frac{\left(Gl\right)}{\left[Gl\right]+K_{A}^{Gl}}\frac{4\left[H^{+}\right]K_{A}^{acid}}{{\left(\left[H^{+}\right]+K_{A}^{acid}\right)}^{2}}, \end{array}$$

with parameter *µ_A_^max^* specifying the maximum rate, *K^Gl^_A_* thehalf-concentration delineating the onset of nutrient uptake saturation, and *K_A_^acid^* the preferred acidity with corresponding pH = −log_10_ *K_A_^acid^*, as shown in Figure 1. Particles of type NA obey the same equation with parameters *µ_NA_^max^*, *K^Gl^_NA_* and *K_NA_^acid^*. The local acidity [*H*^+^] was determined from the acid concentration [*acid*] by using an empirical buffering curve (Strålfors 1948).

Particle growth was tightly linked to sugar metabolism: the increase in particle mass was assumed to be a fixed fraction *Y* of the mass *r* converted from sugar to acid, with the same *Y* for both types. Particles that exceeded a critical diameter after growth divided into 2 nearby daughter particles with the same total mass. High environmental acidity also killed particles at a rate 10^7^ × *K^death^* × [*H*^+^], with *K^death_NA_^* = 2 *K_A_^death^*, which primarily acted near the tooth surface. The biomass was reconfigured after growth, division, and death to ensure mechanical stability.

### System Geometry and Parameterization

The rectangular simulation box was of dimensions *L × L × H*, with the *z*-axis perpendicular to the tooth such that *z* = 0 corresponded to the tooth surface and *z = H* to the air-saliva interface. Constant thickness was maintained by removing particles with *z > B* after mechanical stabilization. Particles were initially placed in the region *0 < z < B*. Further details, color snapshots, and a movie are available in the Appendix.

## Results

### Acidity Profiles Following Sugar Episodes

**Figure 2.**
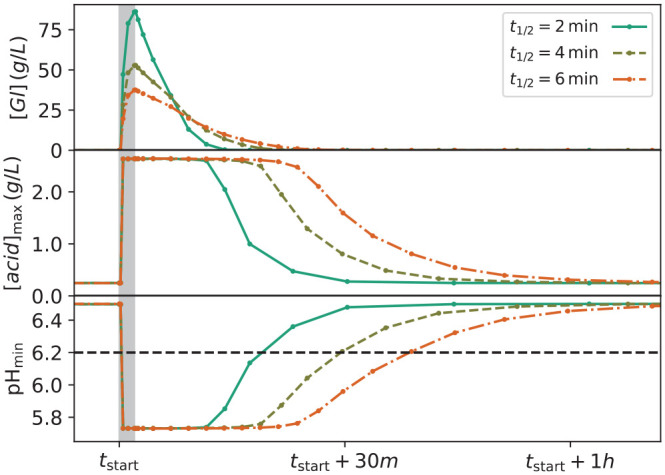
Multipanel plot showing the variation in quantities during a single sugar pulse that started at time *t*_start_. Note that the time axis is shared by all panels. (Top) Sugar concentration [*Gl*] at the saliva-air interface. (Middle) The highest concentration of acid anywhere in the biofilm, [*acid*], metabolized from [*Gl*]. (Lower) The corresponding pH. Curves are plotted for *t*_½_ =  2, 4, and 6 min per the legend. The biofilm composition was symbiotic with 95% NA particles (nonaciduric) and 5% A particles (aciduric). The nominal critical pH for dentin demineralization (pH = 6.2) is highlighted in the lower panel; the same value for enamel (pH = 5.5) is not shown, as such acidity was not achieved for symbiotic biofilms.

The in silico representation of the initial symbiotic (i.e., “health”) dental plaque biofilm was prepared with a predominance of NA particles (nonaciduric; set arbitrarily at 95%) versus A particles (aciduric; 5%). Dietary carbohydrates were introduced to the system by fixing the sugar concentration [*Gl*] at the saliva-air interface according to a predefined profile that rapidly increased to a peak and then decayed exponentially, halving every *t*_½_ (see Fig. 1A). The peak value was chosen for each *t*_½_ so that the integrated concentration of sugars per pulse was constant (Head et al. 2017). The sugar diffused through the saliva into the biofilm where it was partially converted to acid by the particles. Figure 2 shows examples of sugar concentration, acid production, and resultant pH at the tooth surface, for a range of *t*_½_. The acidity increased rapidly and decayed slowly, similar in shape to the applied sugar pulse but with a longer duration. The rapid pH drop and gradual recovery that followed were consistent with known experimental curves (Stephan 1944).

The different molecular composition of dentin leaves it prone to demineralization in more mildly acidic environments than what would be expected to initiate enamel demineralization (Hoppenbrouwers et al. 1987; Shellis 1996). To quantify periods of demineralization, we assumed a critical pH—that is, the pH at which saliva becomes undersaturated with respect to mineral solute—of 6.2 for dentin and 5.5 for enamel, with the understanding that such critical values should be regarded as nominal rather than absolute (Kashket and Yaskell 1992; Dawes 2003). It is clear from Figure 2 that the symbiotic biofilms as parameterized in this study never reached a pH <5.5, from which it may be predicted that enamel demineralization requires a biofilm composition that is markedly dysbiotic in composition. By contrast, the dentin threshold pH of 6.2 was broached for periods exceeding 15 min for all *t*_½_ considered. Also, the time that the pH was below the threshold of 6.2 doubled as the clearance *t*_½_ changed from 2 to 6 min.

### Relationship between Times for Sugar Decay and Acid Clearance

The relationship among the concentration of sugars [*Gl*], the highest concentration of metabolized acid [*acid*], and the corresponding pH was evident when simultaneously plotting the time variation of each quantity during the same pulse (see Fig. 2). Each quantity rapidly changed at the start of the pulse and then relaxed slowly toward prepulse values; however, the profiles for [*acid*] and pH exhibited a plateau extending far beyond *t*_½_, in contrast to the sharp peak in [*Gl*]. This resulted from the dependency of the glycolytic rate on [*Gl*], which saturates for [*Gl*] above *K^Gl^_A/NA_* per equation 1 and Figure 1B. Therefore, acid production for a particle did not significantly decay until the local concentration of sugars around that particle decayed to values below *K^Gl^_A/NA_*. Since *K^Gl^_A_* and *K^Gl^_NA_* were fixed parameters for microbial physiology, the maximum acidity during a pulse did not depend on [*Gl*] whenever it far exceeded *K^Gl^_A,NA_*.

**Figure 3.**
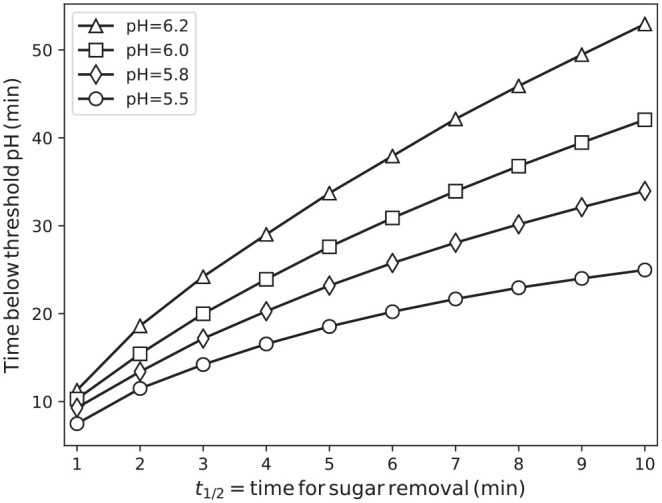
Duration that the pH at the tooth surface was below thresholds ranging from pH 5.5 to 6.2 for different sugar removal times *t*_½_. Data shown for biofilms with 50% NA particles (nonaciduric) and 50% A particles (aciduric). Error bars were much smaller than the symbols.

The key impact of varying *t*_½_ was a change in the duration of the plateau at which the particles’ metabolism remained near saturation and the acidity was near its maximum. Figure 3 shows the time for which the pH passed beyond selected threshold values; *t*_½_ was systematically varied by using a biofilm composition intermediate between symbiosis and dysbiosis, consisting of 50% A particles and 50% NA particles. It is immediately apparent that these times exceeded *t*_½_ by a factor of approximately 5 to 10 over the range considered, although the relationship is not one of strict proportionality, with more gradual increases for longer *t*_½_. This flattening reflects the lower peak sugar concentration for longer *t*_½_ and hence a comparatively shorter time to recross the threshold, required to give the same total sugar input for each pulse per the driving protocol. The curves for lower pH thresholds were also uniformly flatter than those for higher thresholds, reflecting an increased sensitivity to increasing *t*_½_ (slower sugar clearance) when the threshold pH for dentin demineralization (pH = 6.2) is considered, as compared with the threshold for enamel demineralization (pH = 5.5).

### Microbial Ecologic Changes and the Symbiotic-to-Dysbiotic Transition in the Simulated Biofilms

**Figure 4.**
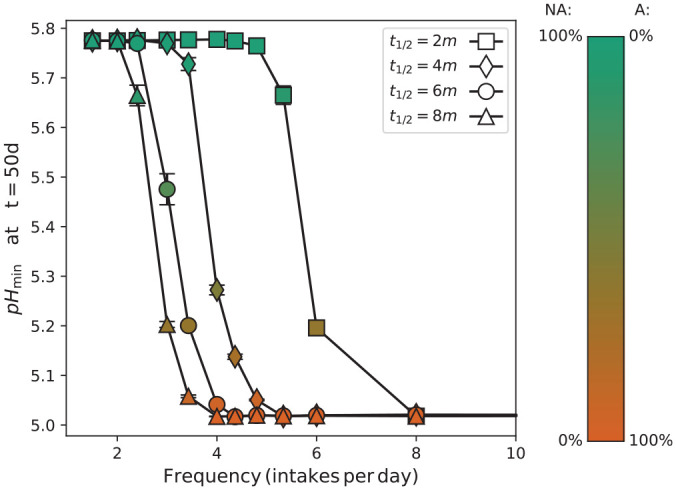
The lowest pH at the tooth surface during a sugar pulse against the frequency of dietary sugar intake, measured after 50 d of growth from a symbiotic composition. Each curve corresponds to the sugar removal time *t*_½_ given in the legend. Symbols have been shaded according to the fraction of A (aciduric) and NA (nonaciduric) particles as per the calibration bar (right).

Predictions for the long-term evolution of dental plaque biofilms and corresponding shifts in potential cariogenicity were made by initializing the system in a symbiotic state as before and applying sugar pulses with fixed *t*_½_ at regular intervals. The 2 particle types, A and NA, were parameterized to grow at different rates depending on the local concentration of sugars and acid, as described in the Materials and Methods section. In particular, particle A grew relatively faster in acidic environments. It was therefore expected that the longer periods of low pH resulting from higher values of *t*_½_ would drive the emergence of dysbiosis. This is confirmed by Figure 4, which shows changes to both the composition and the lowest pH realized during a sugar challenge after 50 d of in silico growth. For a given rate of sugar removal, there was a threshold frequency above which the biofilm composition tended toward a dysbiotic composition dominated by aciduric A particles, and increasing *t*_½_ had the effect of shifting this critical frequency of sugar exposure to lower values. Thus, the longer acid challenges resulting from a reduced rate of sugar removal promoted the growth of dysbiotic biofilms that would generate high acidity near the tooth surfaces. This conclusion did not depend on any specific choice of threshold between symbiotic and dysbiotic compositions, as the entire curves systematically shifted to lower frequencies with increasing *t*_½_.

**Figure 5.**
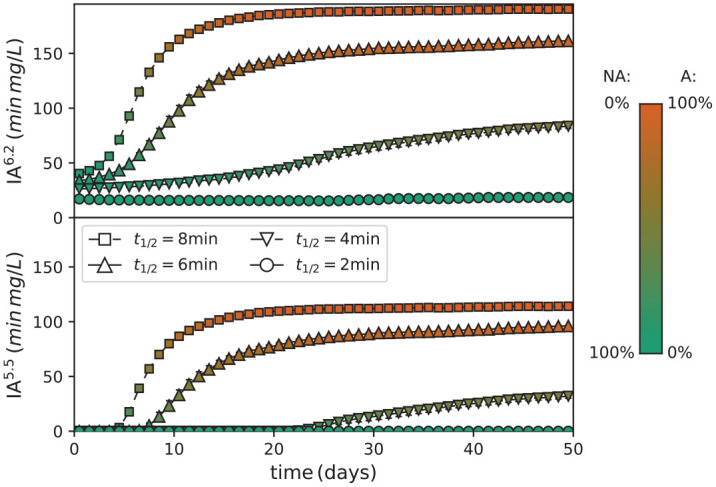
Integrated acid challenge beyond a threshold pH of 6.2 (top) and 5.5 (bottom) for each sugar pulse, as the biofilm composition changes over time. Data correspond to the sugar removal rates *t*_½_ given in the lower legend and 4 pulses per day. Symbol shading reflects biofilm composition per the calibration bar (right).

Acid challenges to dentin or enamel depend not just on the maximum acidity reached during a single episode but also on the duration for which the tooth surface is exposed to the low pH environment. A more sophisticated predictor for cariogenicity than simply the peak acid concentration should therefore integrate acidity over time in some manner. A candidate metric was the integrated concentration of acid near the tooth surface [*H*^+^] measured after dissociation, above some specified threshold for each low-pH episode—that is, the area between the [*H*^+^] curve and the threshold. This quantity was denoted *IA*^6.2^ for a threshold pH of 6.2 relevant to dentin demineralization and *IA*^5.5^ for the corresponding enamel threshold pH of 5.5, both with units of concentration multiplied by time. The evolution in time for both quantities is given in Figure 5 for a range of *t*_½_, demonstrating that the transition to dysbiotic compositions correlated with a significant increase in both these metrics. It is evident from this figure that *IA*^5.5^ was zero for symbiotic compositions, becoming nonzero only after the emergence of dysbiosis for a sufficiently large *t*_½_. By contrast, *IA*^6.2^ was nonzero even for a symbiotic composition, as these pH values can still be produced by nonaciduric NA particles, but nonetheless increased significantly with increasing *t*_½_ as the fraction of A increased.

## Discussion

Aging has a significant impact on oral health; neurogenerative disorders affect oral function and increase the risk for caries (Bakke et al. 2011; Cicciù et al. 2012). In cognitively healthy individuals, the reduction of salivary flow associated with a range of medications increases the overall risk for caries, especially of the more susceptible root surfaces. The present in silico modeling of dentin demineralization and biofilm dysbiosis as a function of prolonged times for sugar clearance (i.e., hyposalivation) clearly highlights major differences in the parameters associated with root versus enamel caries. Prolonging sugar clearance had a major impact on the time for which pH was below the threshold for dentin demineralization, even if a symbiotic biofilm was considered (Fig. 2). As dentin dissolves at higher pH values relative to enamel, the impact of increasing sugar clearance was higher for dentin than for enamel (Fig. 3). These results highlight the increased susceptibility of dentin to demineralization and the deleterious impact of hyposalivation on the dynamics of root caries development; they also offer important metrics in the discussion of the need for more aggressive approaches (e.g., much higher fluoride concentrations) to control root caries when compared with enamel caries (Vale et al. 2011; Fernández et al. 2016; Meyer-Lueckel et al. 2019).

The results also highlight the greater susceptibility of dentin to demineralization under a biofilm that is considered symbiotic—that is, with a low percentage of aciduric species and with a composition that would not affect enamel. Likewise, the higher minimum pH reached in these biofilms would be considered “safe” for enamel but not for dentin dissolution. Therefore, dentin would be susceptible to demineralization under the use of low-cariogenicity sugars, such as lactose or starch, as previously demonstrated experimentally (Aires et al. 2002; Aires et al. 2008; Muñoz-Sandoval et al. 2012), even under normal salivary clearance conditions. Moreover, when this increased susceptibility is associated with a longer period for sugar clearance (i.e., due to hyposalivation), the impact on dentin mineral dissolution is significantly increased, while the longer persistence of low pH levels as salivary clearance is reduced will accelerate biofilm dysbiosis.

The driving force for biofilm dysbiosis is classically associated with an increased frequency of exposure to fermentable sugars, which favors the growth and selection of aciduric species due to the biofilm spending longer periods under low-pH conditions (Bradshaw and Marsh 1998). A strength of in silico modeling is the ability to investigate and compare the influence of different variables independently. In the current study, we started with symbiotic biofilms and assessed the impact of frequency of sugar intake and the sugar removal half-time (*t*_½_) as variables. The model demonstrated that prolonging sugar clearance has a profound effect, converting a symbiotic biofilm to a dysbiotic biofilm at much lower frequencies of sugar exposure per day (Fig. 4). For example, a sugar frequency intake of 3 times per day could be considered “dentally safe” in terms of biofilm dysbiosis (Head et al. 2017) and enamel demineralization under conventional fluoride use (Duggal et al. 2001; Ccahuana-Vasquez et al. 2007). However, if the time for sugar clearance increases from, say, 2 to 8 min due to hyposalivation, then this will result in a lower minimum biofilm pH and thereby promote biofilm dysbiosis.

In this model, integrated acid challenge beyond a threshold pH, rather than time below pH, showed a good correlation with the expected pathogenicity. Figure 5 shows the following for a frequency of exposure to sugar of 4 times per day: 1) high rates of sugar clearance (lower half-times) are not enough to avoid some dentin demineralization, whereas enamel will only start dissolving at much lower rates of sugar clearance; 2) although biofilm dysbiosis is associated with the exposure to sugar and the ability to remove sugar with the resulting acid produced, the effect on the surface, either enamel or dentin, will be substantially different. In other words, a dysbiotic biofilm will cause more damage to dentin than to enamel.

The current model has limitations with regard to the ability to include explicit demineralization reactions and buffering due to demineralization (Kashket and Yaskell 1992; Ilie et al. 2012; Ilie et al. 2014). It has advantages over mathematical models that were 1-dimensional, were designed for a single sugar pulse and not for modeling ecologic changes over longer periods, and did not consider changes to the biofilm composition. To predict the emergence of dysbiosis over prolonged times, the current model reduced computational complexity by employing a single empirical buffering relation and assumed chemical equilibrium at all times. This permitted ecologic predictions to be made in a reasonable computational time. As demonstrated here, the model can be employed to explore the role of relevant parameters/variables on the formation of dysbiotic biofilms and/or the dental tissue. This information can be applied to investigate the role of different therapeutic approaches aimed at preventing root caries. By identifying promising strategies, the model could also help refine the design of clinical studies.

In summary, the current in silico model is applied to study the effect of low sugar clearance rates on dentin (and enamel) demineralization. It can be also used to study how interventions that can influence salivary clearance rates or moderate biofilm pH might prevent bacterial dysbiosis and reduce the prevalence of clinical conditions such as root caries.

## Author Contributions

D. Head, contributed to conception, design, and data analysis, drafted the manuscript; P.D. Marsh, D.A. Devine, L.M.A. Tenuta, contributed to conception, design, and data analysis, critically revised the manuscript. All authors gave final approval and agree to be accountable for all aspects of the work.

## Supplemental Material

sj-pdf-1-jdr-10.1177_00220345211000655 – Supplemental material for In Silico Modeling of Hyposalivation and Biofilm Dysbiosis in Root Caries Click here for additional data file. Supplemental material, sj-pdf-1-jdr-10.1177_00220345211000655 for In Silico Modeling of Hyposalivation and Biofilm Dysbiosis in Root Caries by D. Head, P.D. Marsh, D.A. Devine and L.M.A. Tenuta in Journal of Dental Research
