# Supplementary material for: In Silico Modeling of Hyposalivation and Biofilm Dysbiosis in Root Caries
Source: J Dent Res. 2021 Mar 20;100(9):977–82. doi: 10.1177/00220345211000655 (PMC8293727; doi:10.1177/00220345211000655)
Supplement: sj-pdf-1-jdr-10.1177_00220345211000655 – Supplemental material for In Silico Modeling of Hyposalivation and Biofilm Dysbiosis in Root Caries [file sj-pdf-1-jdr-10.1177_00220345211000655.pdf]

**ONLINE SUPPLEMENTARY MATERIAL** for "In silico modelling of hyposalivation and biofilm dysbiosis in root caries", D.A. Head, P.D. Marsh, D.A. Devine, L.M.A. Tenuta.

## MATERIALS AND METHODS

### System geometry and parameterization

The algorithms used to determine the spatial profiles of concentrations, the parameters for microbial metabolism, and mechanical stability of the biomass are the same as those used previously (Head *et al*., 2014). These and other parameters were taken from a previous application of the same model (Head *et al.*, 2017). The exponential profile for the sugar pulse differed from the square profile employed previously, making it necessary to first ensure that predictions for the range of sugars intake frequencies that resulted in dysbiosis did not significantly change. This was achieved by varying $K^{death}$, $\mu_{A/NA}^{max}$ and $K_{A/NA}^{acid}$ by small amounts until dysbiosis was observed for intake frequencies of ≥4 per day for $t_{1/2} = 4$ min. All other parameters were kept the same. The changes in parameters values resulting from this procedure were never more than 30% from the original values, which we note is much less than the factor of 2 for the variation in specific glucose uptake for two different strains of *S. mutans* (van der Hoeven, 1985), thus we did not regard these changes as significant.

### Color images and movie

Color snapshots for low, intermediate and high values of $t_{1/2}$ are provided in Appendix Fig. 1, and a movie corresponding to a high value of $t_{1/2}$ is provided as Appendix Movie 1.

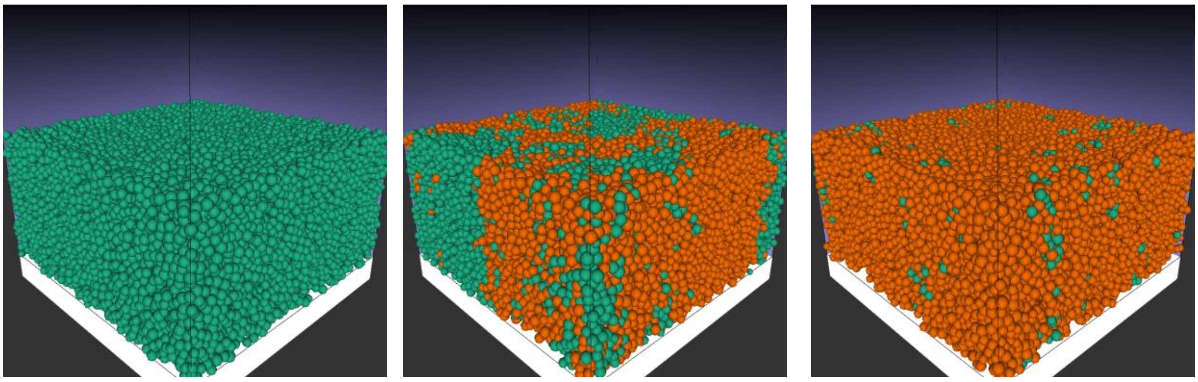

(a) (b) (c)

**Appendix Figure 1.** Snapshots taken after 50 days growth starting from a symbiotic composition of 95% particles of type NA (green), and 5% of type A (red), with sugar removal times $t_{1/2}$ of (a) 2 minutes, (b) 4 minutes, and (c) 6 minutes. The blue in the background corresponds to the undissociated acid, with high (low) brightness for high (low) concentration calibrated to each image separately.

**Appendix Movie 1.** Movie corresponding to the same simulation as Fig. S1(c), showing the transition from an initial symbiotic composition to a dysbiotic one. Filename "MovieS1.wmv".

## REFERENCES

Head, D, Devine, DA, Marsh, PD. 2017. In silico modelling to differentiate the contribution of sugar frequency versus total amount in driving biofilm dysbiosis in dental caries. Sci Rep. 7:17413.

Head, DA, Marsh, PD, Devine, DA. 2014. Non-Lethal Control of the cariogenic potential of an agent-based model for dental plaque. PLOS One. 9(8):e105012.

van der Hoeven, J, Jong, M, Camp, P, van den Kieboom C. 1985. Competition between oral Streptococcus species in the chemostat under alternating conditions of glucose limitation and excess. FEMS Microbiol. Lett. 31(6): 373-379.
